# Low-contrast Pattern-reversal Visual Evoked Potential in Different Spatial Frequencies

**DOI:** 10.18502/jovr.v15i3.7455

**Published:** 2020-07-29

**Authors:** Homa Hassankarimi, Ebrahim Jafarzadehpur, Alireza Mohammadi, Seyed Mohammad Reza Noori

**Affiliations:** ^1^Department of Medical Physics, School of Medicine, Iran University of Medical Sciences, Tehran, Iran; ^2^Department of Optometry, School of Rehabilitation Science, Iran University of Medical Sciences, Tehran, Iran; ^3^Departments of Medical Physics and Biomedical Engineering, School of Medicine, Tehran University of Medical Sciences, Tehran, Iran

**Keywords:** Discrete Wavelet Transform, Fast Fourier Transform, Spatial Frequency, Visual Evoked Potential

## Abstract

**Purpose:**

To evaluate the pattern-reversal visual evoked potential (PRVEP) in low-contrast, spatial frequencies in time, frequency, and time-frequency domains.

**Methods:**

PRVEP was performed in 31 normal eyes, according to the International Society of Electrophysiology of Vision (ISCEV) protocol. Test stimuli had checkerboard of 5% contrast with spatial frequencies of 1, 2, and 4 cycles per degree (cpd). For each VEP waveform, the time domain (TD) analysis, Fast Fourier Transform(FFT), and discrete wavelet transform (DWT) were performed using MATLAB software. The VEP component changes as a function of spatial frequency (SF) were compared among time, frequency, and time–frequency dimensions.

**Results:**

As a consequence of increased SF, a significant attenuation of the P100 amplitude and prolongation of P100 latency were seen, while there was no significant difference in frequency components. In the wavelet domain, an increase in SF at a contrast level of 5% enhanced DWT coefficients. However, this increase had no meaningful effect on the 7P descriptor.

**Conclusion:**

At a low contrast level of 5%, SF-dependent changes in PRVEP parameters can be better identified with the TD and DWT approaches compared to the Fourier approach. However, specific visual processing may be seen with the wavelet transform.

##  Introduction

Contrast is the main issue in visual perception.^[1,2]^ As the first mechanism for visual detection, discrimination and perception may be affected by the contrast level of objects.^[3,4]^ High-contrast objects and symbols are used in the visual examination room as E or similar acuity charts.^[5]^ However, objects in the real world do not show high contrast. Therefore, in the real world, visual function is usually in a low to moderate contrast condition.^[5,6]^ Evaluation of the visual system in a low-contrast situation may indicate its performance in the natural visual environment.

Visual evoked potential (VEP) is a noninvasive and objective electrophysiological test for evaluating human visual function.^[7]^ Pattern-reversal visual evoked potential (PRVEPs) directly mirrors neural activities or the extent of stimulated neural network in each eye using the afferent impulse toward the primary visual cortex (V1).^[8]^ The most prominent and strongest peak in VEP is P100, which has minimal variation and high repeatability. Amplitude and latency of P100 depend on the stimulus conditions, such as the size, luminance, contrast, and spatial frequency (SF).^[9]^ Several studies examining the effects of SF changes on the time domain (TD) parameters of VEP have shown that SF has a significant impact on VEP responses.^[10,11,12]^

Decomposition of the time function into its particular frequencies, amplitudes, and phases by means of the Fourier transform is an objective and common method for the VEP analysis.^[13,14]^ The Fourier technique was successfully applied to determine features of steady-state VEP (SSVEP) and transient VEP (TVEP).^[7,15,16,17,18,19,20,21]^ The power of each frequency band in the frequency domain relates to signal amplitudes in the TD, and phase spectrum provides precise estimation of latencies in TD.^[7]^ The Fast Fourier Transform (FFT) has been employed to measure the VEP phase and amplitude spectrum of the even harmonic response to determine reliability of amplitude and for estimation latency, determine the neural mechanisms in frequency domain, and develop a fast and reliable TVEP technique.^[7,21]^ Zemon et al presented a set of frequency domain measurements that fully obtained the response content and demonstrated that their novel indices may be performed as a more powerful tool to evaluate the visual function. They offered that quantitative and objective measurements in the frequency domain provide a more precise and efficient method for the assessment of the visual system in healthy and diseased brains.^[7]^

The wavelet transform (WT) is a valuable and efficient approach of biosignal processing. This method is widely applied in different studies to analyze, denoise, and extract new parameters of evoked potential signals (EPs), SSVEP, multifocal VEP (mfVEP), TVEP, and PRVEP responses, all of which have totally emphasized on the effectiveness and usefulness of this method.^[22,23,24,25,26,27,28,29,30,31,32,33]^ WT provides simultaneous estimation of time and frequency of VEP signals, which yields noteworthy diagnostic information.^[34]^ Experiments on the SSVEP analysis in time, frequency, and time–frequency domains have suggested that time–frequency and frequency analyses of these waveforms are more efficient than the TD analysis.^[35,36]^

Although several experiments have already evaluated how SF changes affect VEP amplitudes and peak times in TD and VEP amplitude and phase spectrum in the frequency domain, to the best of our knowledge, this issue has not been investigated for the parameters of frequency or time–frequency domains, which are considered in this study. Moreover, the efficiency of the three mentioned dimensions in representing changes of these parameters as a function of SF has not been compared. In the present study, we focused on the relationship between SF and extracted parameters for the PRVEP analysis in time, frequency, and time–frequency domains, and also compared the efficiency of these dimensions in revealing changes.

##  Methods

Thirty-one healthy individuals (19 men and 12 women; mean age, 25.6 ± 6.26 years) participated in this study. All subjects underwent ophthalmic tests and showed a normal visual acuity (minimum and maximum, 0.1 and 0.3 logMAR, respectively). All procedures involving human participants were done in accordance with the ethical standards of the Iran University of Medical Sciences and/or national research committee and with the 1964 Declaration of Helsinki and its later amendments or comparable ethical standards. Written informed consent was obtained from all participants after informing them about the purpose of the study.

Considering the International Society for Clinical Electrophysiology of Vision (ISCEV) protocol, PRVEPs were performed by Metrovision MonPack One (Metrovision Company, Pérenchies, France) with gold-plated cupola electrodes, according to the 10–20 system. Active and reference electrodes were placed on occiput (O${}_{z}$ location) and frontal (F${}_{z}$ location) zones, respectively. The ear lobe served as ground. The PRVEP signals were amplified 2,000 times, filtered in the range of 1–100 Hz and sampled at 1,024 Hz using 240 data points.

A checkerboard pattern alternating at a rate of 2.5 times per second (temporal frequency of 2.5 Hz) was utilized as a stimulus. Test stimuli comprised of spatial frequencies of 1, 2, and 4 cycles per degree (cpd) (corresponding to check sizes of 30, 15, and 7 min of arc, respectively) and contrast level of 5% for each SF. The average sweep numbers per trial was 60.

All VEP waveforms were analyzed in time, frequency, and time–frequency domains using MATLAB software (MATLAB R2015b, The Mathworks, Inc., Natick, Massachusetts, USA). The P100 amplitudes and latencies were evaluated in TD. Following signal normalization, the FFT and discrete wavelet transform (DWT) of P100 peak of all waveforms were carried out in MATLAB environment. MATLAB (matrix laboratory) is a high-level language for high performance numerical computation and visualization. It is an extremely useful, powerful, and popular simulation tool with immense utility in biosignal processing.^[37]^

The FFT and power spectral density (PSD) help determine the frequency components and distribution in fine detail. The mean frequency (F${}_{\mathrm{mean}}$) was derived from FFT, and the mode frequency (F${}_{\mathrm{mod}}$) was extracted from Welch PSD of VEP responses. F${}_{\mathrm{mean}}$ stands for the average frequency in terms of the sampling frequency. F${}_{\mathrm{mod}}$ stands for the most common frequency and refers to the frequency of maximum value in the power spectrum. Hence, F${}_{\mathrm{mod}}$ demonstrates the dominant frequency in the PSD.

**Table 1:** Results of one-way analysis of variance (ANOVA) for three groups of spatial frequency


**Component**	**Group**	Mean ± SD**	**P-value**
	1	6.09 ± 3.49	
Amplitude (µV)	2	3.45 ± 3.1	0.001*
	3	2.529 ± 2.24	
	1	120.9 ± 10.31	
Latency (ms)	2	129.81 ± 15.01	0.021*
	3	131.03 ± 19.35	
	1	8.15 ± 3.85	
F${}_{mean}$ (Hz)	2	7.50 ± 4.92	0.842
	3	8.07 ± 5.30	
	1	2.06 ± 0.36	
F${}_{mod}$ (Hz)	2	2 ± 0.00	0.372
	3	2 ± 0.00	
	1	–4.14 ± 7.27	
Approximation coefficient	2	0.516 ± 8.922	0.005*
	3	2.969 ± 9.24	
	1	–0.914 ± 0.18	
Detail coefficient	2	–0.101 ± 0.205	0.008*
	3	0.0411 ± 0.204	
	1	30.971 ± 16.41	
Descriptor 7P***	2	30.618 ± 15.81	0.051
*Significant difference (*P* < 0.05); **Standard deviation; ***Descriptor of P100 amplitude			

**Figure 1:**
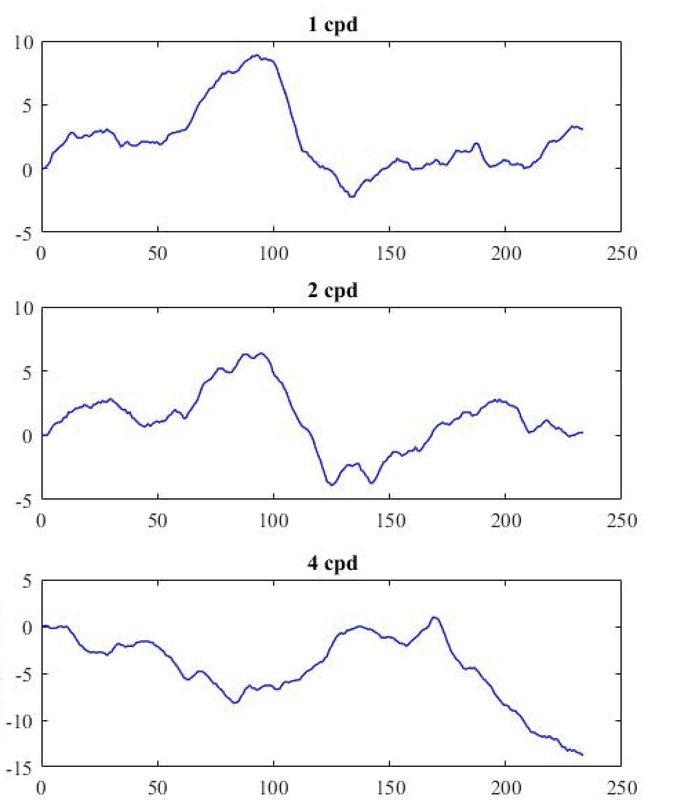
Pattern-reversal visual evoked potentials for one participant at 1, 2, and 4 cpd in the time domain.

**Figure 2:**
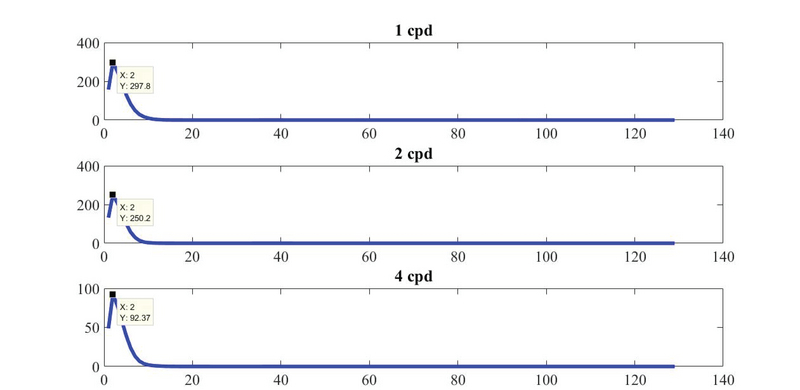
F${}_{\mathrm{mod}}$ extracted from the Power Spectral Density (PSD) of pattern-reversal visual evoked potentials at 1, 2, and 4 cpd. F${}_{\mathrm{mod}}$ is the peak frequency in the PSD (approximately 2 Hz).

The WT is a convolution of frequency contents of the signal (scale) with the wavelet function, which describes a more useful signal information. Discrete wavelet transform decomposes a signal into “detail” coefficients (high-pass filter components) and “approximation” coefficients (low-pass filter components). In DWT, the mother wavelet (Ψ (t)) is decimated by a factor of two and is shifted. The discrete wavelet coefficients can be written as: 

$$\gamma_{j,k}=\int_{-\infty }^{+\infty }x\left(t\right)2^{-j/2}\Psi \left(2^{-j}t-k\right)dt$$

Where integers *j* and *k* represent the scale and shift parameters, and *x (t)* denotes the original signal with the finite length *N*.^[38,39]^

The Daubechies wavelets (Db) are the most important and popular family of wavelets in DWT.^[40]^ With respect to the high resemblance between Daubechies wavelet order of 4 (db4) and PRVEP waveforms, db4 was considered as the proper mother wavelet function for discrete decomposition of PRVEPs in this study.

In all cases, the detail coefficients of levels less than 7 were discarded, as the frequency content of these bands was higher than the P100 frequencies. The approximation coefficients, detail coefficients, and 7P descriptor of all responses in level 7 were computed. The 7P descriptor is the energy percentage of a single wavelet coefficient to the total energy level at predetermined time intervals at level 7 and is extracted from the DWT scalogram. The approach of calculating and extracting the 7P descriptor from the DWT scalograms was previously explained by Hassankarimi et al.^[32]^

All data of TD, FFT, PSD, and DWT were analyzed using the Statistical Package for the Social Sciences for Windows, version 22.0 (Inc., Chicago, IL, USA). After testing the normality of all data, the effect of SF changes on all mentioned parameters was evaluated through a one-way analysis of variance (ANOVA) with the post-hoc Fisher's Least Significant Difference (LSD) test. Spatial frequencies of 1, 2, and 4 (cpd) were considered as groups 1, 2, and 3, respectively.

##  Results

Table 1 shows the results of one-way ANOVA for comparisons of time, frequency, and wavelet domain parameters among three groups of spatial frequencies. For all spatial frequencies, increasing SF resulted in a decrease in the P100 amplitude (Figure 1). The LSD test revealed that differences between 1 and 2 (cpd) groups (*P *= 0.001) and 1 and 4 (cpd) groups (*P *= 0.001) were significant. The latency component also showed a change with increasing spatial frequencies. However, this change was in favor of increasing delay time (Figure 1). Marked latency differences were observed between groups 1 and 2 (*P *= 0.025), and groups 1 and 3 (*P *= 0.011).

Comparing the results of the DWT coefficients revealed that the mean value of both approximation and detail coefficients considerably tended to increase with the increase of SF (Table 1). According to the results of the LSD test, approximation coefficients differed significantly between groups 1 and 2 (*P *= 0.034), and groups 1 and 3 (*P *= 0.001). The increase in these coefficients was much greater by changing the frequency from 1 cpd to 2 cpd than from 2 cpd to 4 cpd. There was a meaningful influence of SF on detail coefficients between groups 3 and 1 (*P *= 0.010) and groups 3 and 2 (*P *= 0.006). The magnitude of the 7P energy descriptor, extracted from the DWT scalograms, showed no observable differences.

The frequency domain components did not change significantly. At all spatial frequencies, the peak frequency (F${}_{\mathrm{mod}}$) had almost a constant value of approximately 2 Hz (Figure 2).

##  Discussion

In the present study, the relationship between SF increase and VEP parameters of time, frequency, and wavelet domains at the contrast level of 5% were investigated. The 5% contrast was considered as the contrast threshold, given the decrease in VEP amplitude due to reduction in contrast and the low signal to noise ratio and negligible VEP responses at the contrast levels < 5%.^[41]^

In agreement with previous studies, our results revealed dramatic changes in TD parameters as a function of SF. An increase in SF resulted in P100 amplitude reduction and latency prolongation (Figure 1).^[11,12,42,43,44]^ It has been supposed that differences in the speed of information processing and conduction along the visual pathways, which are preferentially activated by specific spatial frequencies, lead to the sequential visual processing from low to high range of SFs.^[11]^ Several studies demonstrated that two or more parallel pathways from the retina to the primary visual cortex (V1) are involved in VEP formation. At low contrasts, the magnocellular (MC) pathway dominantly contributes to the VEP responses, whereas at high contrasts, MC, parvocellular (PC), and koniocellular (KC) pathways involve VEP. The MC neurons preferentially detect the low SF and the high temporal frequency stimuli. They have a high contrast sensitivity, high temporal resolution, and short impulse conduction time, whereas PC neurons with a smaller receptive field are sensitive to low temporal and high spatial frequencies.^[1,8,12,35,42,45,46,47,48,49]^ Therefore, MC signals (high SFs) are conveyed to V1 more rapidly than PC signals (low SFs). At high SFs, the optical properties of the eye noticeably reduce the retinal contrast, resulting in decreased amplitude and delayed latency.^[8]^ It has been demonstrated that visual sensitivity progressively weakens with increase in SF or decrease in the size of the object.^[1,50]^ It can be caused by pre-neural factors, such as the optical quality of the eye or by contribution of higher levels of visual processing (beyond the lateral geniculate nucleus) for VEP formation.^[51]^ It is also suggested that quantal fluctuations in light may give rise to sensitivity loss at high SFs.^[52]^

To the best of our knowledge, F${}_{\mathrm{mean}}$ and F${}_{\mathrm{mod}}$ of PRVEPs were not evaluated in previous studies. Our results of the frequency domain analysis showed that changes in SF have no obvious effect on frequency parameters (Table 1). Frequency stability is a significant feature of normal VEP signals. No significant change in F${}_{\mathrm{mean}}$ in all SFs can be explained by the fact that all recorded VEPs in this study were normal. The almost constant value of F${}_{\mathrm{mod}}$ recordings may indicate that, in all groups, VEP responses were generated by the same subsystems and mechanisms. With respect to the stimulus conditions of this study (5% contrast), we conclude that the MC neuron activity dominantly contribute to eliciting the VEPs.^[41]^

Unlike frequency parameters, mean value approximation and detail coefficients represent marked increase as a function of SF (Table 1). Based on the capability of the WT in representing the signal frequency contents locally in time^[53]^ and clear P100 latency prolongation with an increase in SF, considerable differences in time–frequency parameters as a function of SF were expected.

The PRVEPs induced by different SF stimuli originate from segregated neural activities in the visual system. In DWT, approximation coefficients consist of the low-frequency components and the identity of the signal, while the detail coefficients correspond to high-frequency components and fine details of the signal. Statistically significant differences of approximation coefficients between SF groups reflect that the high frequency VEP components at 4 cpd have different origins, generation mechanisms, and visual processing areas compared to other SFs. On the other hand, the low-frequency contents (detail coefficients) at SF of 1 cpd are elicited by different mechanisms compared to the spatial frequencies of 2 and 4 cpd. A possible explanation is that the processing of medium and high SF information occurs in the primary visual cortex (V1), while low spatial frequencies are mainly processed in the secondary visual area (V2).^[1,35]^ Furthermore, SFs > 1.5 cpd generally elicit VEPs that are contrast specific in nature, whereas SFs < 1.5 cpd elicit VEPs that are mainly originated from local luminance changes.^[51]^ Moreover, since the stimulus conditions have a significant impact on the neural responses,^[54]^ under our stimulus conditions, an increase in the DWT coefficients can result in simultaneous stimulation of similar neuronal circuits in the visual cortex and inner cortex interaction between neurons outside the receptive field. As mentioned earlier, MC and PC contribute to VEP response formation. It has been proven that in the 4c layer in V1, the nerve endings projected by MC and PC axon terminals have significant overlapping. Nonselective stimuli activate both magno and parvo systems and give rise to anatomical and functional overlapping.^[51,55]^ In the present experiment, although stimuli contrast was low, selective spatial frequencies had not been chosen specifically to activate the MC neurons. Therefore, neuron activities were not exclusively recorded via VEP responses. Considering the results of the wavelet analysis, it seems that specific SF activates specific receptive field, and, in addition, other factors are also involved in the response formation mechanisms.

In summary, the obtained results indicate that optical information processing is performed through parallel pathways in the visual system. In addition, the visual system can select a dedicated channel for processing of specific information according to different optical properties. This system has distinct spatial and contrast filters, and this filtration is associated with stimulus condition.

In conclusion, the authors evaluated the SF effect on PRVEP features in time, frequency, and time–frequency domains and concluded that the TD and DWT approaches are more efficient compared to the FFT and PSD approaches to detect the impact of SF on the VEP parameters at a contrast level of 5%. Furthermore, sources, mechanisms, and pathways involved in evoking and processing PRVEP responses are SF dependent. We suggest further research on more subjects with stimuli of different contrasts, using other wavelet functions.

##  Financial Support and Sponsorship

Nil.

##  Conflicts of Interest

There are no conflicts of interest.

##  Appendix

### MATLAB Codes

#### Code for normalizing the input

%Normalization range is [-2 2]

function [normalized_output] = Normalization(input)

temp03 =.5 * (max(input) + min(input));

temp04 =.5 * (max(input) – min(input));

input = 2 * (input - temp03) / temp04;

t05 = isnan(input);

input(t05) = 0;

t06 = input > 2;

input(t06) = 2;

normalized_output = input;

#### Time domain analysis

X = p;

Y = t;

Z = x;

Fs = 1024;       % Sampling frequency

T = 1/Fs;       % Sampling period

L = 240;       % Length of signal

t = (0:L-1)*T*1000;       % Time vector

subplot(3,1,1);plot(t,X,'b');

subplot(3,1,2);plot(t,Y,'b');

subplot(3,1,3);plot(t,Z,'b');xlabel('time(ms)');ylabel('amplitude (µV)');

#### Frequency domain analysis and power spectral density 

X = d;

Fs = 1024;       % Sampling frequency

T = 1/Fs;       % Sampling period

L = 240;       % Length of signal

tm = (0:L-1)*T*1000;      % Time vector

subplot(2,2,1);plot(tm,X,'r');

Y = fft(X);

P2 = abs(Y/L);

P1 = P2(1:L/2+1);

fr = Fs*(0:(L/2))/L;

subplot(2,2,2);plot(fr,P1);

title('Single-Sided Amplitude Spectrum of X(t)')

xlabel('f (Hz)')

ylabel('|P1(f)|');

M = meanfreq(X,Fs);

pxx = pwelch(X);

subplot(2,2,3);plot(pxx);xlabel('f (Hz)');

#### Calculate approximation and detail coefficients of Discrete Wavelet Transform 

data = Normalization(b);

wname = 'db4'; % Wavelet Mather Functidn Name

nLevel = 7; % Wavelxt Decomposition Level

[C, L] = wavedec(data,nLevel,wname);% Wavelet Decomposition

A7 = appcoef(C,L,wname,nLevel);    % Approximation Coefficients

D7 = detcoef(C,L,7);       % Detail Coefficients of Level 7

D6 = detcoef(C,L,6);       % Detail Coefficients of Level 6

D5 = detcoef(C,L,5);       % Detail Coefficients of Level 5

D4 = detcoef(C,L,4);       % Detail Coefficients of Level 4

D3 = detcoef(C,L,3);       % Detail Coefficients of Level 3

D2 = detcoef(C,L,2);       % Detail Coefficients of Level 2

D1 = detcoef(C,L,1);       % Detail Coefficients of Level 1

#### Calculate d7 descriptor

d7 = D7(1, 6)∧2;

power_D7 = sum(D7.∧2);

pd7 = d7/power_D7 *100;
